# Significantly higher faecal counts of the yeasts *candida* and *saccharomyces* identified in people with coeliac disease

**DOI:** 10.1186/s13099-017-0173-1

**Published:** 2017-05-05

**Authors:** Joanna Harnett, Stephen P. Myers, Margaret Rolfe

**Affiliations:** 1 Faculty of Pharmacy, The University of Sydney, Camperdown, Australia; 2 Division of Research, Southern Cross University, Sydney, Australia; 3 University Centre for Rural Health, School of Public Health, University of Sydney, Sydney, Australia

**Keywords:** Coeliac disease, Microbiota, Yeasts, *Candida*, *Saccharomyces*

## Abstract

**Background:**

Coeliac disease is an autoimmune disorder resulting from an interaction between diet, genome and immunity. The treatment of CoeD is lifelong adherence to a gluten free diet, which is associated with clinical and histological improvements. However, a substantive number of individuals report only partial symptom improvement despite both compliance with a strict gluten free diet and improvements in serological and histological biomarkers of disease activity. The role of the intestinal microbiota is an area of interest in this sub-group.

**Aims:**

To investigate the role of yeasts and parasites in individuals reporting persistent symptoms of Coeliac disease (CoeD).

**Methods:**

Forty-five people who met the ESPGHAN diagnostic criteria for CoeD were recruited via the Australian Coeliac Association. The faecal measures of the DNA of yeasts and parasites from the CoeD group were compared to data obtained from the medical records of non-coeliac controls with gastrointestinal symptoms from other causes.

**Results:**

*Candida* sp. was detected in 33% of the CoeD group compared 0% of the control group (p = 0.000) and *Saccharomyces* sp. was detected in 33% of the CoeD group compared to 10% of the control group (p = 0.026). There were no differences in the presence of any of the parasite species measured.

**Conclusion:**

Further research is required to understand the significance of C*andida* and *Saccharomyces* species in both the aetiology of CoeD and of persistent symptoms in this sub-group.

*Trial Registration* Clinical Trial Registration—ANZCTR Number: 12610000630011

## Background

Coeliac disease (CoeD) is an autoimmune disorder resulting from an interaction between diet, genetic factors and immunity. The global prevalence of CoeD is estimated to be 1% with a significant number of people remaining undiagnosed. Coeliac disease can present at any age with a broad range of intestinal and extra-intestinal symptoms [1]. However, anaemia, diarrhoea and fatigue remain the most common symptoms at presentation [1]. The strict adherence to a gluten free diet (GFD) is an effective treatment in most cases however a sub-group of patients report the effectiveness of dietary treatment may reduce over time or they attain only partial symptom improvement [2, 3]. The role of the intestinal microbiota in the aetiology of the disease and/or as a cause of partial symptom improvement in a sub-group of patients with CoeD is an area of interest [4].

There is a growing body of evidence to suggest that the composition of the intestinal microbiome is associated with a number of chronic diseases including obesity [5], diabetes, [6] inflammatory bowel disease, [7] and bowel cancer [8]. The composition of the gastrointestinal microbiome of children and infants with CoeD has also been investigated in a number of studies [9]. The results of these studies have identified significant differences in the numbers and diversity of gastrointestinal bacterial species in the microbiota of infants and children with CoeD compared to healthy children [10–16]. In adults reporting persistent symptoms of CoeD, alterations in the composition of the gastrointestinal microbiota have been reported despite their long-term adherence to a gluten free diet [17]. To our knowledge, neither parasitology nor mycology been included in any microbiota composition studies of people reporting persistent symptoms or only partial symptom improvement. Understanding the causes of partial symptom improvement is a clinically relevant area of CoeD research due to the association with a reduced quality of life [3]. We hypothesised that a sub-group of adults with CoeD reporting only partial symptom improvement despite adherence to treatment of a strict gluten free diet (GFD) will have an altered faecal microbial composition with an increased presence of yeasts and parasites compared to non-CoeD individuals reporting gastrointestinal symptoms from other causes.

## Methods

### Study design

This study was nested into a clinical trial that has been reported elsewhere [18]. The study sample consisted of 45 people with CoeD reporting mild to moderate gastrointestinal symptoms and an external control group who had undertaken the same faecal testing used in this study, at the same laboratory, and within the same time period who also reported gastrointestinal symptoms from other causes but were negative for CoeD.

#### Ethics

The study was approved by the Human Research Ethics Committee of Southern Cross University (ethics approval number ECN-12-021). The research was conducted in compliance with good clinical practices (GCP) and in accordance with the guidelines of the Australian National Health and Medical Research Council and the Declaration of Helsinki (as revised in 2004). ANZCTR Number: 12610000630011.

#### Recruitment, inclusion criteria and clinical characteristics of the CoeD group

This study used the Australian Coeliac Association’s (ACA) diagnostic criteria/definition for CoeD which is based on the updated version of the 1990 ESPGHAN criteria for CoeD. The CoeD group (n = 45) was recruited through a cohort of survey respondents sent via the Australian New South Wales CoeD Association email database.

Inclusion criteria were: (a) between 18 and 70 years of age; (b) CoeD confirmed by small bowel biopsy greater than 12 months prior to enrolling in the study; (c) were adhering to a gluten free diet (GFD) for at least 12 months; d) were not pregnant; e) were not diagnosed with major gastrointestinal pathology (e.g. cancer or inflammatory bowel disease); (f) had recent bowel or oral surgery; (g) were not HIV-positive; (h) did not have active alcohol and or illicit drug dependence; (i) had not taken non-steroidal anti-inflammatory drugs, or antibiotics in the 4 weeks prior to undertaking faecal testing; and (j) had no clinical abnormalities in serum urea, electrolytes, creatinine or liver function values at baseline testing.

CoeD participants were interviewed by the study coordinator (JH) who took a medical history, screened for adherence to a GFD and classified gastrointestinal symptom scores obtained using a baseline validated Coeliac Disease Questionnaire (CDQ) [19]. The results of the CDQ analysis has been discussed in detail elsewhere [18]. Brief details of the CDQ are provided for completeness. The CDQ was employed to establish the severity of symptoms. The CDQ consisted of four sub-categories including emotion (including fatigue as a symptom), worries, social and gastrointestinal symptoms (urgency to defecate, loose bowel motions, abdominal cramping, bloating or flatulence, incomplete bowel evacuation, repeated belching, nausea or retching). The gastrointestinal symptoms were classified by responses to 7 questions with a 7 point Likert-scale response rating severity. The total gastrointestinal symptom sub-scale scores could range from 0 to 49. Lower scores are associated with more severe persistent symptoms (0–21) and are more likely to be associated with CoeD activity and/or other pathology. Scores of 22–35 were considered mild to moderate and scores 42–49 were considered normal. In addition, the comprehensive clinical assessment at baseline included questions that would identify red flags of gastrointestinal pathology including unexplained weight loss, blood in the stool or black stools. All participants were questioned about whether they had consulted their doctor and/or gastroenterologist about their gastrointestinal symptoms. Fatigue was also considered a persistent symptom. Fatigue scores at baseline were mild to moderate.

#### Recruitment, inclusion criteria and clinical characteristics of external control group data

The control data in this trial was obtained from patient test records. A single medical practitioner held this data. The microbial ecology profile in these cases had been ordered on the basis of symptoms suggesting irritable bowel syndrome and the need to exclude other causes including parasitosis, bacterial infection; and to determine if there was significant dysbiosis underlying the symptoms. In addition, all of the control group participants had negative Coeliac serology and normal sIgA levels. The process conducted in obtaining access to these records was in accordance with a protocol that had obtained human research ethics committee approval. The study coordinator (JH) was granted access to patient test result records but not clinical progress notes. The first 50 individuals with faecal microbial results who were also proven to be serologically negative to CoeD and over the age of 18 were contacted by mail. A letter providing information and requesting permission to use their test result data, accompanied by a consent form, was sent to the 50 individuals meeting the study criteria. Twenty-seven individuals returned a signed consent form and these individuals’ faecal microbial results formed the non-CoeD control data group.

Inclusion criteria for the control group were (a) men and women aged 18–75 years; (b) had undertaken DNA faecal analysis in the previous 2 years; (c) who had tested serologically negative to tissue transglutaminase (tTg) and anti-gliadin IgA in the previous 12 months and; (d) individuals with normal blood sIgA.

#### Participant collection of faecal specimens

The CoeD participants were provided specimen collection instructions: faecal specimen samples were collected in 50 ml conical tubes containing Formalin, culture and sensitivity media and nucleic acid extraction buffer. The participant refrigerated the faecal samples immediately after collection. The specimens were collected from the participant by a biological courier and delivered to the study site. After the study coordinator (JH) checked the specimens for temperature and time stability they were shipped on ice to Metametrix Laboratory (Atlanta, Georgia, USA) who undertook the testing. Faecal specimens were processed between one and three days of receipt for DNA extraction and sensitivity assays, and microscopy was performed on the tubes containing formalin. The stool specimens were stored at 4 °C following processing and discarded 30 days from the accessioning date.

### Outcome measures

A primary outcome measure was the semi-quantitative measures of yeasts and the detection of parasites in faecal samples obtained from the faecal microbial analysis. Detection was made using polymerase chain reaction amplification of the DNA of each organism reported. The laboratory method is described by Scott et al. [20].

*Candida* sp. and *Saccharomyces* sp. and non-specified yeasts were reported by the laboratory as +1, +2, +3 or +4 indicating >100, >1000, >10,000 or 100,000 parts per gram (pg) of DNA per gram (g) of faeces respectively.

*Parasites* were reported as detected or not detected. Faecal specimens were assessed for the presence of *Blastocystis hominis, Dientoemba fragiilis, Nector americanus, Trichuris trichuria, Enterobius vermicularis, Entaeomba histolytica, Entaeomba* sp., *Cryptosporidium* sp., *Endolimax nana, Giardia* sp., *Trichomonas hominis, Ascaris lumbricoides* (Round worm)*, Clonorchis sinensis* (Chinese liver fluke worm)*, Schistosoma mansoni, Strongyloides* sp., and *Taenia solium* (Tape worm).

All CoeD participants’ faecal results were forwarded, with the participants’ permission, to their nominated doctor with a covering letter and information about the study. All control group data was managed clinically by the medical practitioner who had ordered the faecal test as part of patient management.

### Statistical analysis

A sample size calculation was undertaken by the methodologist and statistician at Southern Cross University. The sample size was estimated using PASS 2008™ sample size soft-ware. The statistical packages used were SPSS PASW^®^Statistics GradPack 18 and version 20 SPSS. Significance was assumed if p ≤ 0.05.

Yeasts were reported by the laboratory on a semi-quantitative count scale and parasites were reported as detected or not detected. The semi-quantitative counts of *Candida* and *Saccharomyces* species in faecal samples were reported as positive +1, +2, +3 or +4 indicating >100, >1000, >10,000 or >100,000 parts DNA per gram of faecal specimen. Chi square analysis was conducted to ascertain the statistical differences between groups. Significance was assumed at p ≤ 0.05.

Chi square analysis were conducted to ascertain whether there were any statistical differences between the two groups in semi-quantitative yeast counts. The number and percentage for each microorganism was recorded. Parasite detection data were analysed by conducting exact Chi square tests on the two-way contingency Tables (CoeD/non-CoeD and detected/not detected).

All authors had access to the study data and reviewed and approved the final manuscript.

## Results

### Demographics

A total of 37 females and eight males (mean age 47.3 years) residing in New South Wales, Australia, were allocated to the CoeD arm of this study. A total of 20 females’ and 7 males’ (mean age 44.5 years) faecal microbial ecology profile results and Coeliac serology formed the final control data set. The gastrointestinal symptom scores were mild to moderate in all cases except two cases who scored higher than 42 but were lower in the symptom of fatigue, therefore warranting their inclusion. Fatigue is a common persistent symptom of CoeD.

### Results of primary outcome measure—yeasts and parasites

A significant difference in the detection of *candida* species was found (p = 0.000) between groups. No candida was detected in the faecal specimens of the control group compared to 33.3% of the CoeD specimens. A significant difference in the detection of *saccharomyces* was also found (p = 0.026) between groups. These results are presented in Table 1. In all cases where *saccharomyces* sp. and/or *candida* sp. were reported as detected, the semi-quantitative measures determined were 2+, i.e.>10,000 parts per gram (pg) of DNA per gram (g) of faeces on a scale that goes from 1 to 4+.

**Table 1:** Mycology: results of Chi square analysis, number and percentage distribution and p values for comparison of detection of yeasts between COED Group and Control Group (the cut-off level for reporting the detection of yeast was >10, 000 parts per gram of DNA per gram of faeces)

Yeast species	CoeD group n = 45		Control group n = 27		Chi square p value Fisher’s exact test
	Not detected n (%) <10,000 parts per gram of DNA per gram of faeces	Detected n (%) 2+ >100,000 parts per gram of DNA per gram of faeces	Not detected n (%) <10,000 parts per gram of DNA per gram of faeces	Detected n (%) 2+ >100,000 parts per gram of DNA of faeces	
Yeast taxonomy unavailable	27 (60)	18 (40)	16 (59.3)	11 (40.7)	0.572
*Candida* sp.	30 (66.6)	15 (33.3)	27 (100)	0 (0)	0.000
*Saccharomyces* sp.	30 (66.6)	15 (33.3)	24 (89.3)	3 (10.7)	0.026

Parasitology results are presented in Table 2 showing the number, percentage distribution and p-values of Chi square analysis. The DNA of non-human parasites with an unknown taxonomy were detected at a significantly higher rate in the CoeD group compared to the control group however no other significant differences were found between groups for specific species of the parasites looked for. The following parasites were not detected in either group; *Entaeomba histolytica, Entaeomba* sp., *Cryptosporidium* sp., *Endolimax nana, Giardia* sp., *Trichomonas hominis, Ascaris lumbricoides* (Round worm)*, Clonorchis sinensis* (Chinese liver fluke worm)*, Schistosoma mansoni, Strongyloides* sp., and *Taenia solium* (Tape worm).

**Table 2:** Parasitology: results of Chi square analysis, number and percentage distribution and p values for comparison between detection rates of parasites in CoeD and control group (parasite DNA was reported simply as detected or not detected)

Parasite Name	CoeD group n = 45		Control group n = 27		Fisher’s exact test (Chi square p value)
	Not detected n (%)	Detected n (%)	Not detected n (%)	Detected n (%)	
General parasite incidence	4 (8.9)	41 (91)	5 (21)	22 (78.6)	0.168
Parasite taxonomy unavailable	5 (11.1)	40 (88.9)	7 (28.6)	20 (71.4)	0.058
*Blastocystis hominis*	37 (82.2)	8 (17.8)	22 (82.2)	5 (17.9)	0.614
*Dientoemba fragilis*	43 (95.6)	2 (4.4)	23 (85.7)	4 (14.3)	0.147
*Nector americanus*	42 (93.3)	3 (6.7)	27 (100)	0 (0)	0.228
*Trichuris trichuria*	43 (95.6)	2 (4.4)	27 (100)	0 (0)	0.377
*Enterobius vermicularis*	41 (91.1)	4 (8.9)	23 (85.7)	4 (14.3)	0.363

## Discussion

### The potential significance of the faecal yeast *Candida* sp. in CoeD

The human mycobiome (fungi and their genome) is a relatively new advance in characterising the residents of healthy individual’s gastrointestinal tracts. One gastrointestinal mycobiome characterisation study identified 66 fungal genera and 184 fungal species, with *Candida* as the dominant fungal genera [21]. Fungi have been associated with a number of gastrointestinal diseases, with a dominant focus on the mycobiome of patients with IBD and graft-versus-host disease [21].

The clinical significance of increased numbers of commensal yeasts is not fully understood in those other than individuals who are critically ill or immune compromised. To our knowledge this is the first study reporting statistically significant differences between the prevalence of faecal *Candida* sp. counts of people with CoeD reporting persistent symptoms compared to people without CoeD. However, in vitro, animal and clinical studies have identified potential mechanisms of action as to the causal relationship between *candida* and CoeD and these will be discussed briefly here. The role of candida in triggering aberrant immune response to dietary proteins has been supported by an animal study that demonstrated that gastrointestinal *Candida* colonisation promotes sensitisation against food antigens, partly due to mast cell mediated hyperpermeability in the gastrointestinal mucosa [22]. Dysregulation of the normal integrity of the small intestinal mucosa, i.e. small intestinal hyperpermeability is a feature of the pathophysiology of CoeD [23, 24]. An association between an intestinal candida infection in those genetically predisposed CoeD has been proposed as a potential trigger of the disease process [25, 26]. This association was first described by Nieuwenhuizen et al., who hypothesised that the virulence factor of *C. albicans*-hyphal wall protein 1 (HWP1) contains amino acid sequences that are identical or highly homologous to known CoeD-related α-gliadin and γ-gliadin T cell epitopes [26]. The HWP1 is used by *C. albicans* to adhere to the intestinal epithelium. It is thought that tTg and endomysium components link to the yeast and act as an adjuvant that activates the immune system to fight the HWP1 and gluten, thereby forming autoimmune antibodies against tTg and endomysium, resulting in the characteristic villous atrophy of CoeD [26]. More recently, Courage et al. proposed that the common denominator in the humoral cross-reactivity observed between HWP1 and gliadin in both CoeD and candida infection was transglutaminase further suggesting that candida infection could be a trigger of CoeD in the genetically predisposed [25].

Other research groups have identified at least three species of *Candida* sp. that produce proteases that can degrade immunoglobulin-A1 (Ig-A1), Ig-A2 and sIgA. Interestingly, of 2098 patients with CoeD in one study, 2.6% had an sIgA deficiency, representing a 10–16-fold increase over that of sIgA deficiency in the general population [27]. Furthermore, it has been shown that sIgA deficiency may be a predisposing factor to autoimmune diseases [28], and to recurrent infections [29]. In this present study, there were no cases of sIgA deficiency in the non-CoeD control group. However, we did not have data on the levels of the sIgA from the CoeD group at point of diagnosis nor did we measure sIgA as the focus of this study was on the role of the microbiota as cause of persistent symptoms not as an aetiological factor. Nevertheless, the detection of measureable counts of *Candida* sp. in 33% of the CoeD group compared to 0% in the control group (p = 0.000) raises the question; can *Candida* sp. act as an immunosuppressant through an ability to reduce sIgA, thus allowing colonisation of the intestine and triggering autoimmune responses such as CoeD in the genetically predisposed? The findings from this study and those from the studies discussed above warrants further research that explores the role of *Candida* sp. and the relationship between *Candida* and sIgA and the onset of CoeD.

A possible link between *Candida albicans* and the aetiology of the inflammatory bowel disease (IBD) Crohns disease has been reported [30]. *Candida* taxa have been found in increased abundance in the faceal microbiota of children with IBD compared to children without IBD with the authors concluding that it is important to explore the fungal microbiota as playing a possible role in the pathogenesis of the disease [31]. In a mouse model study, *Candida albicans* colonisation was found to augment dextran sulphate sodium induced inflammation and conversely inflammation was strongly promoted *Candida albicans* colonisation [32]. A possible interpretation of this finding as it relates to CoeD could be that the chronic small intestinal inflammation characteristic of CoeD, is potentially induced by intestinal *Candida albicans* colonisation thus contributing to the pathophysiology of the disease. In established CoeD, intestinal *Candida albicans* colonisation may augment and promote inflammation resulting in only partial clinical and/or histological improvements.

While *Candida* sp. may or may not be a fungal environmental trigger for CoeD, this study found that *Candida* sp. was more prevalent in the faeces of people with mild to moderate persistent symptoms of CoeD compared to those with gastrointestinal symptoms from other causes. Antibiotics have a profound impact on the composition of the composition of the gastrointestinal microbiome and are considered the most common cause of opportunistic colonisation by *C. albicans.* As part of the inclusion criteria for this study no participant in the CoeD group had taken antibiotics within 4 weeks of undertaking testing. However, they did report a history of recurrent antibiotic use as children, i.e. >once per year in the first 12 years of life. This is higher than estimated use by the general western population where it has been estimated that half of the paediatric population of most Western countries receive antibiotics at least once per year [33]. Antibiotic use in childhood has been implicated as potential trigger for CoeD onset [34]. In this present study we did not gather data regarding the specific proximity of antibiotic use and CoeD onset due to the difficulty in determining the duration of CoeD prior to a medical diagnosis. In line with the discussion regarding the opportunistic growth of *candida* sp being secondary to antibiotic administration, is the important consideration that the increased prevalence of these yeasts could also be secondary to the altered architecture and function of the small intestine that is characteristic of CoeD. Therefore, it is theoretically possible that as the small intestine recovers to normal structure and function that the increased counts of yeasts could be self-limiting. However, should opportunistic yeast overgrowth of the small intestine be identified as secondary to the pathophysiology of CoeD in future studies, small intestinal yeast overgrowth in addition to small intestinal bacterial overgrowth can be considered in those failing to respond to dietary treatment.

### The potential significance of the faecal yeasts *Saccharomyces* sp. in CoeD

To our knowledge, it has not been reported previously that people with CoeD have a significantly higher prevalence of *Saccharomyces* sp. counts in their faeces compared to those without CoeD. A potential confounding factor in the interpretation of this finding could be related to dietary ingestion and transient numbers of *S. cerevisiae* being detected in the faeces due to its prevalent use in the manufacturing of wine, beer and bread [35]. Anti-*S. cerevisiae* antibodies (ASCAs) have been found in 43% of patients with CoeD at diagnosis and these antibodies disappeared during treatment with a GFD [36]. The disappearance of ASCAs after treatment was found to be more pronounced in children than in adults. It is suggested that ASCAs are more likely to persist in treated adult CoeD patients due to the more profound damage of the intestinal wall (as a consequence of their delayed diagnosis); therefore, resolution of intestinal permeability is slower [36]. This present study’s findings would suggest that treated adult CoeD patients with persistent symptoms have higher indigenous faecal counts of *Saccharomyces* sp. and the presence of ASCAs may not be secondary to intestinal permeability, but rather a suggestion that there is colonisation. *Saccharomyces boulardi* is commonly prescribed as a probiotic supplement for individuals with gastrointestinal symptoms such as diarrhoea and has attracted research attention by many groups for its therapeutic potential [37, 38]. None of the CoeD group reported were taking *s. boulardi* prior to this baseline test. The potential clinical implications of taking supplemental probiotic formulations of *s. boulardi* in these individuals is not known. CoeD patients who present to their health care professionals with persistent symptoms or partial symptom improvement despite adherence to a GFD are often faced with further invasive and non-invasive investigations and are subject to a dietary audit. If these investigations yield no explanation for their symptoms they are sent home with a range of pharmaceuticals that may provide symptomatic relief. This study indicates that gastrointestinal dysbiosis is prevalent in this population and faecal assessment may provide important clinical information in this sub-group of CoeD patients.

### Study limitations

The addition of ASCAs as a biomarker would have strengthened the design of this study and provided greater insight as to the potential clinical relevance of the molecular detection of *saccharomyces* in faecal samples of indications with CoeD. Future studies, exploring this question further are encouraged to include ASCAs as a biomarker in evaluating this question.

There are a number of limitations for studies exploring components of the microbiome of specific populations including this study. Firstly, there are only general microbial markers of what constitutes a healthy intestinal microbiome and there is much ambiguity around the clinical significance of alterations in the measures of specific commensal microbial residents. In light of this, our findings are potentially limited by the fact we did not use a healthy control group. This present study employed control data obtained from a group of heterogeneous individuals who attended a doctor with symptoms consistent with irritable bowel syndrome and had excluded CoeD as part of their medical assessment. Conversely, while this may be deemed as a limitation, it may also be interpreted as strength in further differentiating groups of people troubled by gastrointestinal symptoms.

Secondly, sub-speciation and employing Sabroud culture methods of the yeasts detected may have provided more clinically significant information. Lastly, there is currently no gold standard for the molecular assessment of the intestinal microbiota with each technique having strengths and limitations that may result in discrepancies between findings and result bias. This study may have been strengthened through having the faecal specimens analysed by two laboratories to verify the findings.

Given the limitations of this study we cannot conclude there is a relationship of cause and effect but instead seek to report an important observation. We strongly encourage larger scale, rigorously designed clinical studies to further investigate these findings.

## Conclusion

The increased prevalence of the yeasts *Candida* sp. and *Saccharomyces* sp. in CoeD patients reporting only partial symptom improvement has not been reported before and these findings warrant larger scale studies specifically designed to explore the significance of these organisms as both a cause of persistent symptoms in people adhering to a GFD and in the aetiology of the disease. If these results are supported by future studies, it could aid in the development of appropriate antifungal or probiotic interventions.

## Abbreviations

ACA: Australian Coeliac Association
ASCAs: anti-*saccharomyces cerevisiae* antibodies
ANOVA: analysis of variance
CDQ: Coeliac Disease Questionnaire
CoeD: coeliac disease
CFUs: colony forming units
GA: georgia
GEE: Generalised Estimated Equations
GFD: gluten free diet
IBD: inflammatory bowel disease
IL: interleukin
IFN: interferon
NA: not available
NATA: National Association of Testing Authorities
NSW: New South Wales
RCT: Randomised Controlled Trial
TGF: transforming growth factor
TNF: tumour necrosis factor
UAC: unable to calculate
